# A Study of Proton Pump Inhibitors and Other Risk Factors in Warfarin-Associated Gastrointestinal Bleeding

**DOI:** 10.7759/cureus.12624

**Published:** 2021-01-11

**Authors:** Jevon Tang, Umesh Sharma, Shreya Desai, Janos Molnar, Lawrence Perlmuter, Axel Feller, Pallavi Shah

**Affiliations:** 1 Gastroenterology, Captain James A. Lovell Federal Health Care Center, North Chicago, USA; 2 Internal Medicine, Chicago Medical School/Rosalind Franklin University of Medicine and Science, North Chicago, USA; 3 Cardiology, Chicago Medical School/Rosalind Franklin University of Medicine and Science, North Chicago, USA

**Keywords:** drug-drug interactions, proton-pump inhibitor, bleeding risks, warfarin, gastrointestinal bleeding

## Abstract

Background

Warfarin users are at increased risk for gastrointestinal bleeding (GIB). History of GIB, stroke, cardiovascular or chronic kidney disease, age greater than 65 years, and drug interaction with proton pump inhibitors (PPI) have previously been identified as risk factors for GIB in warfarin users. We hypothesized that concomitant use of warfarin and PPI would increase the incidence of GIB relative to warfarin use alone.

Methods

We did a retrospective review of medical records of 626 patients taking warfarin for at least two weeks. Parameters including age, concomitant medication use (non-steroidal anti-inflammatory drugs (NSAID), aspirin, selective serotonin reuptake inhibitors (SSRIs), PPI, and anti-platelet drug), history of GIB, chronic renal failure (CRF), and peptic ulcer disease (PUD) prior to warfarin use were analyzed.

Results

Variables that increase the likelihood of bleeding in warfarin users included aspirin, PPI, history of PUD, history of previous GIB, CRF, and elevated prothrombin time (PT)/international normalized ratio (INR) values. Concomitant antiplatelet use showed a slight increase in GIB but this was not statistically significant (p=0.082). NSAID use and SSRI use were not associated with a higher risk of GIB among warfarin users. Patients who are on PPI and warfarin simultaneously are more likely to be on acetylsalicylic acid (ASA) or have a history of PUD, GIB, or CRF, all of which are associated with increased incidences of GIB.

Conclusions

Although concomitant use of warfarin and PPI appears to be associated with an increased incidence of GIB, these patients are more likely to have other risk factors that also increase the risk of a GIB outcome. Therefore, the interaction between PPI and warfarin is clinically insignificant.

## Introduction

Warfarin is used annually by millions of Americans because of prosthetic heart valves, chronic or paroxysmal atrial fibrillation, recurrent deep venous thrombosis, hypercoagulable states, and vascular diseases. Warfarin is an antagonist of vitamin K that is required in the synthesis of clotting factors II, VII, IX, and X, and endogenous anticoagulant proteins C and S. The incidence of hemorrhage during anticoagulant therapy ranges from 0.8% to 10.6% [1-3]. It carries a mortality rate as high as 67% if the hemorrhage involves the brain [4]. However, the gastrointestinal tract remains the most common focus of hemorrhage [5,6]. Because warfarin is metabolized by the liver, patients who are taking multiple medications metabolized by the liver are at an increased risk for developing complications while on warfarin. 

Proton pump inhibitors (PPI) are a class of medications that are thought to have significant interactions with warfarin since they both utilize the same cytochrome p450 for clearance [7], and therefore, significantly increase the risk of hemorrhage [8]. However, clinical studies have failed to demonstrate this consistently [9,10]. Several confounding factors such as diet, concomitant use of other medications, compliance with warfarin use, and international normalized ratio (INR) monitoring were proposed unsuccessfully to explain these discrepancies. This issue became particularly important in our geriatric population as most elderly patients, especially those in a nursing home, are on multiple medications and often PPI and warfarin are among those medications. Therefore, we studied the effect of concomitant PPI and warfarin use, and other confounding factors, on gastrointestinal bleeding (GIB).

## Materials and methods

Data collection

We performed a retrospective review of medical records of 626 male patients at the North Chicago Veteran Affairs Medical Center (NCVAMC), who were identified from the pharmacy database as having received prescriptions for warfarin for at least two consecutive weeks between the years 2000 and 2005 and had otherwise been free of GIB for six months prior to entering the study. Variables, including age, concomitant medication use (non-steroidal anti-inflammatory drugs (NSAID), aspirin, selective serotonin reuptake inhibitors (SSRIs), PPI, and anti-platelet drug), history of GIB prior to warfarin use, history of chronic renal failure (chronic renal failure (CRF); serum creatinine > 1.5 mg/dl) and history of peptic ulcer disease (PUD) were collected by three trained researchers. GIB was defined as a hemoglobin drop of ≥2g/dl presenting as hematemesis and/or melena or other lab evidence suggesting blood loss from the gastrointestinal tract resulting in a referral to the gastroenterology service. Laboratory data including prothrombin time (PT) and INR were also collected. For subjects with GIB, the PT and INR values at the time of GIB were recorded; for non-bleeders, two consecutive highest INR and PT values were used. 

Statistical analyses

Data analysis was performed using Statistical Package for Social Sciences (SPSS) software, version 10 (SPSS Inc, Chicago, IL). Continuous variables were analyzed by independent sample t-tests. Categorical variables were analyzed by the chi-square test. Logistic regression analysis was performed to evaluate predictor variables for GIB. Differences were considered statistically significant with a two-sided p-value of <0.05.

## Results

The participants in our study comprised of older male veterans. The mean age of patients in the GIB group while on warfarin was 74.79 years while the mean age of patients in the non-GIB group was 72.52 years. The p-value was not statistically significant between the two groups (p=0.142). We did not stratify the results based on gender due to all participants being male in this study. 

Our multiple logistic regression analysis revealed that GIB was significantly associated with aspirin use, history of GIB, CRF, and a higher level of PT (Table 1). In addition, subgroup analysis using paired t-test showed the increased incidence of GIB in patients treated with PPI. However, NSAID use (p=0.816), and SSRI use (p=0.165) were not associated with a higher risk of GIB among warfarin users (Table 2). PT and INR were significantly lower in the group who were on a PPI than the group who were not on a PPI (Table 3).

**Table 1:** A comparison of study variables in patients with concomitant use of both warfarin and NSAIDs versus warfarin alone PT, prothrombin time; INR, international normalized ratio; NSAID, non-steroidal anti-inflammatory drugs; PUD, peptic ulcer disease; SSRI, selective serotonin reuptake inhibitors; *, statistically significant.

	NSAID use	No NSAID use	P values
N	139	489	
Mean Age	70.78	73.31	0.038*
PT	26.76	27.48	0.448
INR	2.44	2.54	0.262
New GI Bleed	13 (9.4%)	49 (10%)	0.816
Use of Aspirin	39 (28.1%)	103 (21.1%)	0.08
Use of PPI	57 (41%)	178 (36.4%)	0.322
History of PUD	9 (6.5%)	31 (6.3%)	0.954
Prior history of GI bleed	7 (5%)	29 (5.9%)	0.689
Use of SSRI	22 (15.8%)	53 (10.8%)	0.109
History of Chronic Renal Failure	18 (12.9%)	73 (14.9%)	0.559

**Table 2:** Comparison of comorbidities in warfarin users with and without GIB GIB, gastrointestinal bleeding; PT, prothrombin time; INR, international normalized ratio; NSAID, non-steroidal anti-inflammatory drugs; PUD, peptic ulcer disease; SSRI, selective serotonin reuptake inhibitors; *, statistically significant Risk ratios and confidence interval for the risk of gastrointestinal (GI) bleeding in warfarin-treated patients analyzed in relation to clinical history (Age, GI bleeding in the past), co-morbidities (chronic renal failure (CRF), gastroesophageal reflux disease (GERD), PUD), concomitant use of other medications (aspirin, SSRI, proton pump inhibitors (PPI), Anti-platelet agents, NSAIDs) and abnormal lab results (elevated PT, INR). History of GI bleeding, aspirin use, CRF, and elevated PT in warfarin-treated patients favor re-bleeding. *p<0.05, **p<0.02, ***p<0.001.

	Presence of GI Bleed	Absence of GI Bleed	P Values
Number of patients (N)	62	566	
Mean Age	74.79	72.52	0.142
PT value	32.47	26.75	0.009*
INR value	2.86	2.48	0.049*
Use of NSAID	13 (21%)	126 (22.3%)	0.816
Use of Aspirin	24 (38.7%)	118 (20.8%)	0.001*
Use of Anti-Platelet medications	10 (16.1%)	52 (9.2%)	0.082
Use of PPI	34 (54.8%)	201 (35.5%)	0.003*
History of PUD	8 (12.9%)	32 (5.7%)	0.048*
Prior History of GI Bleed	29 (46.8%)	7 (1.2%)	<0.001*
Use of SSRI	4 (6.5%)	71 (12.5%)	0.165
History of Chronic Renal Failure	23 (37.1%)	68 (12%)	<0.001*

**Table 3:** A break down of studied variables in patients who use both warfarin and PPI concomitantly versus warfarin alone PPI, proton pump inhibitors; GI, gastrointestinal; PT, prothrombin time; INR, international normalized ratio; NSAID, non-steroidal anti-inflammatory drugs; PUD, peptic ulcer disease; SSRI, selective serotonin reuptake inhibitors; *, statistically significant.

	PPI use	No PPI use	P values
N	231	390	
PT	25.9	28.15	0.006*
INR	2.41	2.58	0.019*
New GI Bleed	34(14.5%)	28(7.1%)	0.003*
Use of Aspirin	79(33.6%)	63(16%)	0.001*
History of PUD	28(11.9%)	12(3.1%)	0.001*
Prior history of GI Bleed	22(9.4%)	14(3.6%)	0.002*
History of chronic renal failure	54(23%)	37(9.4%)	<0.001*
Use of NSAIDs	57(24.3%)	82(20.9%)	0.322
Use of Anti-Platelet medications	37(15.7%)	25(6.4%)	<0.001*
Use of SSRI	32(13.6%)	43(10.9%)	0.317

Through logistic regression analysis, we found that the previous history of GIB was the strongest predictor of GIB in patients who were on warfarin. This was followed by aspirin use, history of CRF, and PT (Table 4). None of the other factors we examined (e.g., age, NSAIDs, anti-platelet agents, SSRI, and history of PUD) can be considered to be independent risk factors.

**Table 4:** Logistic regression analysis with the following variables entered: NSAID use, ASA use, anti-platelet use, PPI use, SSRI use, CRF CRF, chronic renal failure; PPI, proton pump inhibitors, ASA, acetylsalicylic acid; PT, prothrombin time; INR, international normalized ratio; NSAID, non-steroidal anti-inflammatory drugs; PUD, peptic ulcer disease; SSRI, selective serotonin reuptake inhibitors; *, statistically significant.

	(p)
N	628
Age	.839
PT	.045*
INR	.224
History of PUD	.134
Prior history of GI bleed	< .001*
Use of NSAIDs	.859
Use of Aspirin	.018*
Use of Anti-platelets	.312
Use of Proton pump inhibitor	.308
Use of SSRIs	.065
History of chronic renal failure	.039*

## Discussion

This study shows that the incidence of gastrointestinal (GI) bleed in warfarin users increased with ASA use, previous history of GIB, CRF, and a higher level of PT. Compared to patients taking warfarin only, simultaneous use of PPI and warfarin is strongly associated with new GIB, however, the significance is not seen in the multiple regression model. There is possible drug-drug interaction between PPI and warfarin leading to an increased warfarin concentration because PPI and warfarin both utilize the cytochrome p450 for clearance [7]. However, monitoring warfarin concentration is difficult and does not correlate to clinical outcomes. Two patients with the same warfarin concentration may have different PT and INR values because warfarin acts on multiple steps in the clotting cascade. Therefore, clinicians measure INR to monitor the effectiveness of warfarin.

Previous studies reported that increased INR levels are positively associated with an increased incidence of GIB [11-13], and similar results are seen in our study between warfarin users with GIB and those that do not have GIB (Table 1). However, patients who were using PPI and warfarin concomitantly had a lower INR, but an increased incidence of GIB, than those who were only taking warfarin. This suggested that the drug interaction between PPI and warfarin might not be the reason for an increased GIB, but other risk factors might have contributed to the increased risk of GIB. In our study, the use of ASA, history of PUD, GIB, or CRF were identified as predictors for new GIB, and we found that these conditions were more common in patients who used PPI and warfarin concomitantly than warfarin alone (Table 3). 

On the other hand, the lower INR in the patients who were using PPI and warfarin concomitantly than warfarin alone did not translate to a lower incidence of GIB, although the protective role of PPI in GIB is well described [14]. One explanation is that the protective effect of PPI is limited to the stomach where acid is produced, whereas warfarin use and other co-morbid conditions increase the incidence of GIB in the entire gastrointestinal tract. ASA itself is known to cause peptic ulcer disease that can lead to GIB [15], which is prevented by PPI [14]. On the other hand, patients with CRF have an increased incidence of arteriovenous malformation (AVM) that could result in both upper and lower GI bleed [16,17]. In addition, distal small bowel and colonic ulcers, and colon cancers may result in lower GIB. PPI primarily reduces stomach acid production and decreases the bleeding due to stomach ulcers, but gastrointestinal lesions at other parts of the gastrointestinal tract have the potential to bleed under warfarin stress.

In our study, anti-platelet agents and NSAID, which are also known to cause gastric ulcers and GIB [18], did not show a statistically significant increase in the risk of GIB when used concomitantly with warfarin. However, it is important to point out that in a cohort study performed in Japan including 17,270 patients, the incidence of NSAID induced bleeding was reported to be only 0.05% [19]. NSAID users in our study were significantly younger patients which could possibly explain the decreased incidence of GIB in the NSAID group [6,20]. Other factors that are not examined in our study but were suggested in the literature, such as more frequent PT and INR monitoring because physicians are aware of the potential risk of bleeding with concomitant warfarin and NSAID use, and a lower dosage of NSAID received by patients who are taking warfarin concomitantly, have contributed to the similar bleeding outcome between patients who use warfarin and NSAID concomitantly and patients who use warfarin alone [21,22].

It was shown that there is a weak link between SSRI and upper GIB at a population level in patients who are not taking warfarin [23-27] as SSRI impair platelet aggregation [26]. This is most significant in patients who were using SSRI and NSAID concomitantly. When using SSRI alone, the risk of bleeding is low [28]. However, in our study, we found that SSRI use is not a risk factor for GIB in warfarin users.

While our study did not evaluate the effects of direct oral anticoagulants (DOAC) on GI bleed risk, we would like to highlight the recent data available. A previous meta-analysis has shown that while DOACs reduced the risk of strokes, intracranial bleeds, and mortality, the risk of GI bleed increased [29].

Additionally, we would like to comment on a recent meta-analysis evaluating the risk of upper GIB among oral anticoagulants and PPI co-therapy [30]. PPI co-therapy was shown to decrease the incidence of GI bleed across all anticoagulants including warfarin [30] which is contrary to our study. The authors showed warfarin users on PPI co-therapy had reduced incidence of hospitalizations for their GI bleed. However, their subgroup analysis looking at GI bleed score showed that the subgroup who was on PPI co-therapy had a higher GIB risk score. The GI bleed risk score was calculated using multiple variables including advanced age, previous history of GI bleed, current use of medications that increase risk of GI bleed, cardiovascular risk, and past history of GI emergency department visit [30].

Limitations

Our study is a retrospective analysis and has several inherent limitations including exclusive reliance upon older male veterans. Despite these limitations, the present study may provide some insights for further research into the risk factors of GIB in warfarin users.

## Conclusions

We provided some evidence that only a history of GIB, ASA use, CRF, and an elevated PT may be identified as independent risk factors for GIB in warfarin users. Moreover, the interaction between PPI and warfarin is clinically insignificant with respect to GI bleeding. While PPI is given to prevent future GIB in patients who are on aspirin, anti-platelet medications, NSAID, or to patients with a history of peptic ulcer disease or history of GIB, their primary sites of action are in the stomach, and their effect on bleedings from other sites is uncertain. SSRIs, which cause an increase in upper GIB in NSAID users, do not cause an increased incidence of GIB in warfarin users. While the current guidelines do not require more frequent INR monitoring in warfarin users who are at increased risk of bleeding, studies have shown that more frequent monitoring may lead to a longer duration of INR in the therapeutic range, which in turn is associated with fewer adverse effects. We should study the cost-effectiveness of more frequent INR monitoring in warfarin users presenting with a history of GIB or ASA use or CRF to prevent GIB.
